# Neuromodulation of Cerebral Blood Flow: A Physiological Mechanism and Methodological Review of Neurovascular Coupling

**DOI:** 10.3390/bioengineering12050442

**Published:** 2025-04-23

**Authors:** Jiawen Zhong, Gen Li, Zexiang Lv, Jingbo Chen, Chunyan Wang, Ansheng Shao, Zhiwei Gong, Junjie Wang, Siqiao Liu, Jun Luo, Shuping Yang, Sibei Wu, Lin Ning, Zhinong Wang, Jiahao Li, Yu Wu

**Affiliations:** 1 School of Pharmacy and Bioengineering, Chongqing University of Technology, Chongqing 400054, China; zhongjw2024@163.com (J.Z.); lzx17354355169@163.com (Z.L.); ansheng20000224@163.com (A.S.); gongzhiweicq@163.com (Z.G.); wangjunjie0511@163.com (J.W.); liusiqiao0504@163.com (S.L.); junxiao0119@gmail.com (J.L.); ysp20191844309@163.com (S.Y.); wusibei0908@163.com (S.W.); linning0625@163.com (L.N.); wangzlcq@163.com (Z.W.); lijiahao25800314@163.com (J.L.); 2 Faculty of Naval Medicine, Naval Medical University, Shanghai 200433, China; jimmychen777@smmu.edu.cn

**Keywords:** neurovascular coupling, cerebral blood flow, multimodal techniques

## Abstract

Neurovascular coupling (NVC) refers to the dynamic regulation of cerebral blood flow via neuronal activity, a mechanism crucial for maintaining normal brain function. This review elucidates the intricate physiological mechanisms underlying NVC, emphasizing the coordinated roles of neurons, glial cells, and vascular cells in mediating activity-induced changes in blood flow. We examine how NVC is impaired in neurological disorders such as Alzheimer’s disease and stroke, where the dysfunction of this coupling contributes to neurodegeneration and neurological deficits. A broad range of techniques for assessing NVC is discussed—encompassing the established modalities like transcranial Doppler, near-infrared spectroscopy, and functional magnetic resonance imaging (fMRI), as well as emerging technologies such as functional ultrasound imaging and miniaturized endoscopy that enable high-resolution monitoring in deep brain regions. We also highlight the computational modeling approaches for simulating NVC dynamics and identify the novel biomarkers of NVC dysfunction with potential utility in early diagnosis. Finally, emerging translational applications—including neuromodulation techniques and targeted pharmacological interventions—are explored as means to restore normal neurovascular function. These advancements underscore the clinical significance of NVC research, paving the way for improved diagnostic tools and therapeutic strategies in neurological disorders.

## 1. Introduction

Neurovascular coupling refers to the regulation of the local cerebral blood flow via neuronal activity through a series of intercellular signaling pathways to meet the metabolic demands of neurons [1]. The relationship between neuronal activity and regional cerebral blood flow was first observed in open skull experiments at the end of the 19th century [2]. This discovery boosted functional brain mapping, adding optical methods to neurographic recording. An extension to closed skull studies, applicable to humans, started with Positron Emission Tomography (PET) [3]. With the support of these technological and foundational studies, the physiological phenomenon of neurovascular coupling has been explored in greater depth. Studies show that neurovascular coupling is crucial in maintaining normal brain function. In cerebrovascular diseases, abnormalities in the neurovascular coupling mechanisms can lead to the dysregulation of cerebral blood flow, subsequently causing neurological damage [4,5,6,7]. In Alzheimer’s disease, the dysfunction in neurovascular coupling is closely associated with neuronal decline. In addition to the aforementioned diseases, neurovascular coupling dysfunction is also closely associated with vascular dementia and cerebral small vessel disease. These conditions are characterized by impaired cerebral autoregulation, chronic hypoperfusion, and progressive cognitive decline [8,9]. Given their clinical importance and their direct relevance to neurovascular coupling dysfunction, these diseases deserve further in-depth exploration. However, due to space limitations, this review does not provide a detailed discussion of these conditions and instead focuses on Alzheimer’s disease and stroke. A deeper exploration of the regulatory mechanisms of neurovascular coupling not only helps reveal the physiological nature of cerebral blood flow regulation but also provides important theoretical support for the early diagnosis, intervention, and personalized treatment of cerebrovascular diseases [10,11,12].

This review summarizes the physiological mechanisms of neurovascular coupling and its implications for clinical disorders. We discuss the fundamental anatomical structures, signaling pathways, and regulatory processes of cerebral blood flow associated with neurovascular coupling. Additionally, we review the current major assessment techniques, including transcranial Doppler (TCD), near-infrared spectroscopy (NIRS), functional magnetic resonance imaging (fMRI), and multimodal techniques. We also provide a perspective on two emerging techniques: functional ultrasound (fUS) and miniaturized endoscopy, discussing their potential applications in future neurovascular coupling assessments. With the development of multimodal technologies, integrating these assessment methods enables researchers to comprehensively understand the regulatory mechanisms linking neural activity and cerebral blood flow, thereby providing more precise insights for clinical diagnosis and treatment. Finally, this paper explores the future development of neurovascular coupling research from three perspectives: the underlying mechanisms, new detection concepts, and clinical applications, to provide theoretical support for the clinical research applications in this field.

## 2. Physiological Mechanisms of Neurovascular Coupling

### 2.1. The Link Between Neuronal Activity and Blood Flow Supply

Neurovascular coupling refers to the dynamic regulatory mechanism between neuronal activity and local cerebral blood flow. The brain has a high metabolic demand for oxygen and glucose. When neuronal activity in a specific brain region increases, the local blood flow rapidly increases to meet these metabolic needs, thus maintaining metabolic homeostasis [13]. The brain’s blood supply primarily relies on the internal carotid artery (ICA) and the vertebral artery (VA), both of which originate from the subclavian artery. Approximately 70% of the cerebral blood flow is provided by the internal carotid artery, while the vertebral arteries supply the remaining portion. The vertebral arteries merge to form the basilar artery, which, as part of the circle of Willis, branches to supply the brainstem, cerebellum, and occipital cortex [14,15,16,17]. During neuronal activity, the consumption of local oxygen and glucose increases, and the neurovascular coupling mechanism regulates vasomotion by sensing the changes in the local metabolic byproducts, ensuring that metabolically active regions receive an adequate blood flow [18,19].

**Figure 1:**
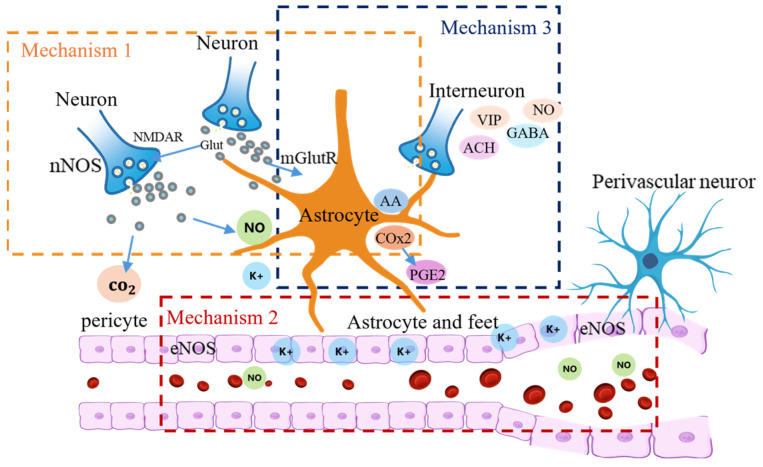
The three mechanisms by which neurovascular coupling regulates cerebral blood flow.

There are three main mechanisms through which neurovascular coupling regulates the cerebral blood flow, as illustrated in Figure 1 [20]. The first mechanism involves the electrical activity of neurons inducing neurotransmitter release, which acts on endothelial cells to regulate vasoconstriction and vasodilation. The second mechanism involves astrocytes sensing neuronal activity and releasing regulatory factors that indirectly influence endothelial and smooth muscle cells to regulate the cerebral blood flow. The third mechanism involves endothelial cells sensing changes in local signaling molecules, which act on smooth muscle cells to directly participate in vascular constriction and dilation [21].

### 2.2. Intercellular Signal Transmission

Neurovascular coupling relies on the coordinated actions of multiple cell types, including neurons, astrocytes, endothelial cells, vascular smooth muscle cells, and pericytes, which together form the functional neurovascular unit (NVU). This functional unit bridges the gap between neuronal activity and cerebral blood flow regulation, ensuring the efficient delivery of energy and oxygen to meet the metabolic demands of neurons [22].

**Figure 2:**
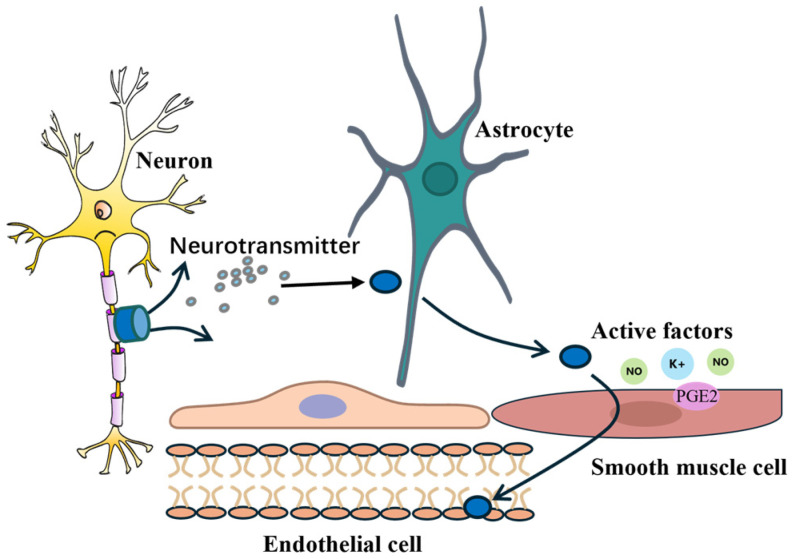
Schematic illustration of intercellular signaling within the neurovascular unit.

Neurons serve as the fundamental structural and functional units of the brain and are responsible for receiving, integrating, and transmitting signals. Neurons exhibit various morphologies, with a basic structure consisting of a cell body, dendrites, and an axon [23]. Dendrites receive external stimuli, while axons transmit electrical impulses along established pathways to synaptic terminals, where neurotransmitters are released. Astrocytes detect neuronal activity through membrane-bound receptors and respond by releasing a variety of active factors (prostaglandins, nitric oxide, and potassium ions), which act on endothelial cells and vascular smooth muscle cells to induce vasodilation or vasoconstriction. Endothelial cells play a critical role in modulating the permeability of the vascular wall and integrating the signaling pathways involved in local cerebral blood flow regulation. Vascular smooth muscle cells, in turn, are directly responsible for the dynamic changes in the vessel diameter. This complex and highly coordinated intercellular signaling process is illustrated in Figure 2, which depicts the interactions among neurons, astrocytes, endothelial cells, and smooth muscle cells. Neurons release neurotransmitters that activate astrocytes, which subsequently secrete vasoactive factors acting on the vasculature to modulate the regional cerebral blood flow [24].

### 2.3. Synergistic Action of Signaling Molecules

Neurovascular coupling relies not only on the coordination between cerebral blood flow and neuronal activity but also on the fine regulation of various signaling molecules. Multiple signaling molecules mediate the connection between neuronal activity and blood supply through distinct pathways. Nitric oxide (NO), prostaglandin E2 (PGE2), and calcium ions (Ca^2+^) are the most critical regulatory factors in neurovascular coupling. When neurons are excited, the levels of these molecules increase, leading to vasodilation and an increase in the local blood flow [25].

NO is synthesized by endothelial nitric oxide synthase (eNOS) and neuronal nitric oxide synthase (nNOS) and can diffuse into vascular smooth muscle cells. It activates soluble guanylate cyclase (sGC), promoting the production of cyclic guanosine monophosphate (cGMP), which induces vasodilation and increases the local blood flow. PGE2 is primarily released by astrocytes and endothelial cells, acting on specific receptors on vascular smooth muscle cells, leading to vasodilation. Additionally, PGE2 can enhance the effects of NO, further amplifying the impact of neuronal activity on the blood supply. Ca^2+^ directly regulates the function of endothelial cells and vascular smooth muscle cells. During neuronal excitation, Ca^2+^ concentrations rise, promoting the release of glutamate molecules, which activate astrocytes and further stimulate the release of NO and PGE2 [26].

It is worth noting that the synergistic interactions among these signaling molecules exhibit variations across different brain regions and developmental stages. On one hand, regional heterogeneity in cellular composition, metabolic activity, and vascular density contributes to the spatial specificity of neurovascular regulation. For instance, the prefrontal cortex and hippocampus differ in the structure and function of neuronal, glial, and vascular units, leading to region-specific mechanisms of signaling molecule modulation. Studies have shown that PGE_2_ plays a more prominent role in regulating the cerebral blood flow in the cortical areas, whereas NO predominates in neurovascular coupling within the hippocampus. Additionally, Ca^2+^-dependent signaling pathways appear to be more responsive to rapid hemodynamic changes in the visual cortex [27,28].

On the other hand, the efficiency and molecular basis of neurovascular coupling undergo dynamic changes from early development through aging. During the early developmental stages, astrocytes are not yet fully matured, limiting the synthesis and release of PGE_2_, thereby making NO a more dominant factor in vascular regulation. In contrast, aging is associated with endothelial dysfunction, the reduced expression of nNOS, and diminished efficiency of calcium-signaling pathways, all of which may impair the synergistic actions of these molecules and compromise the dynamic regulation of the local cerebral blood flow. Age-related microvascular remodeling may further alter the diffusion patterns and targets of signaling molecules, resulting in changes in their spatiotemporal effects [29,30,31].

## 3. Neurovascular Coupling Impairment in Neurological and Cerebrovascular Diseases

Neurovascular coupling is a key mechanism in cerebral blood flow regulation, ensuring the coordination between neuronal activity and the local cerebral blood flow. Dysfunction in this mechanism impairs the regulation of the cerebral blood flow and may lead to insufficient metabolic supply to neurons, ultimately resulting in neurological damage. Studies have shown that neurovascular coupling impairment plays a significant role in various diseases (Table 1). However, the mechanisms by which different diseases affect neurovascular coupling vary [32]. To gain a deeper understanding of the mechanisms and clinical significance of neurovascular coupling dysfunction, the following sections will focus on two representative diseases: Alzheimer’s disease and stroke. Alzheimer’s disease, as a typical neurodegenerative disease, has pathological features closely associated with neurovascular dysfunction. In contrast, a stroke directly impacts the cerebrovascular system, causing acute damage to neurovascular coupling [33].

### 3.1. Mechanisms of Neurovascular Coupling Impairment

**Figure 3:**
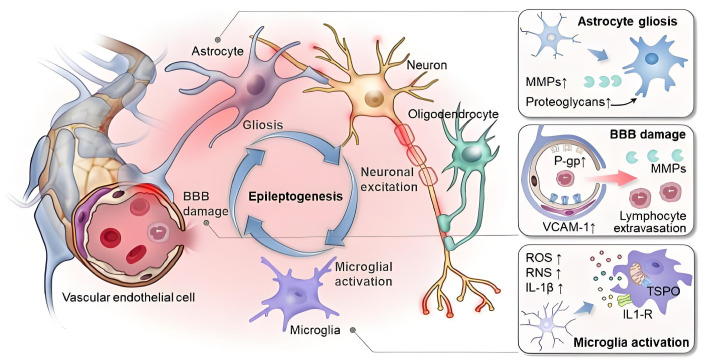
The disruption of neurovascular unit homeostasis leads to molecular, functional, and structural abnormalities [45].

When the homeostasis of the neurovascular unit is disrupted, it can lead to the onset of diseases, as shown in Figure 3. Endothelial dysfunction is an important factor contributing to neurovascular coupling dysregulation. Endothelial cells regulate vasodilation by releasing molecules such as NO, and damage to the endothelial cell function inhibits this process, leading to the abnormal regulation of the cerebral blood flow. Secondly, the dysfunction of astrocytes is another critical factor in the impairment of neurovascular coupling. Astrocytes regulate the cerebral blood flow through calcium signaling and the production of cyclic adenosine monophosphate. In various cerebrovascular diseases, the function of astrocytes is affected to different extents, reducing their role in neurovascular coupling and, consequently, affecting cerebral blood flow regulation. Furthermore, changes in neuronal function, such as the increased excitability of neurons or excessive release of excitatory neurotransmitters like glutamate, can disrupt the balance of neurovascular coupling. Particularly in neurodegenerative diseases such as Alzheimer’s disease, excessive excitatory neuronal activity not only directly damages neurons but also exacerbates the dysfunction of neurovascular coupling [8,42,43]. In addition, chronic inflammation and oxidative stress play significant roles in various neurological diseases. Inflammatory factors alter the regulatory mechanisms of neurovascular coupling, increasing the instability of the local cerebral blood flow and leading to further neural damage [44].

### 3.2. Impairment of Neurovascular Coupling in Alzheimer’s Disease

Alzheimer’s disease is a progressive neurodegenerative disorder primarily characterized by the gradual loss of cognitive function and behavioral disturbances. Research indicates that the onset of Alzheimer’s disease is closely associated with the decline in neurovascular coupling function. Patients with Alzheimer’s disease typically exhibit a reduced cerebral blood flow and disrupted neurovascular coupling, particularly in the cortical and hippocampal regions. An important mechanism underlying the neurovascular coupling impairment in Alzheimer’s disease is the disruption of the blood–brain barrier (BBB) [46]. Under normal conditions, the BBB maintains the exchange of substances between the cerebral blood flow and neurons through tight junctions in endothelial cells. However, in Alzheimer’s patients, this barrier function is compromised, leading to a reduced ability to regulate the local blood flow in brain regions. The neurotoxicity caused by β-amyloid deposition leads to neuronal hyperexcitability and enhanced inflammatory responses, resulting in impaired vascular function and further disruption of neurovascular coupling [47,48,49]. Notably, in Alzheimer’s patients, a weakened vasodilation response is often observed, meaning that the local cerebral blood flow fails to increase appropriately with heightened neuronal activity, leading to neuronal hypoxia and inadequate metabolic supply, thus increasing the risk of cognitive impairment [50,51,52]. Furthermore, the role of astrocytes in Alzheimer’s disease is also compromised. β-amyloid deposition in Alzheimer’s not only directly affects the neuronal function but also alters the astrocytic function. Astrocytic calcium signaling is inhibited, diminishing their role in neurovascular coupling and, consequently, affecting the regulation of the local cerebral blood flow.

### 3.3. Impairment of Neurovascular Coupling After Stroke

A stroke is an acute neurological injury caused by the interruption of the cerebral blood flow, which can be classified into ischemic and hemorrhagic. Regardless of the type of stroke, the impairment of neurovascular coupling is closely linked to changes in the cerebral blood flow and often has a profound impact on the recovery of neurological function in patients [53].

In the early stages of ischemic stroke, a significant reduction in the cerebral blood flow leads to local neuronal hypoxia and metabolic disturbances, thereby affecting the normal function of neurovascular coupling. The reduction in the cerebral blood flow suppresses the necessary blood flow increase required by neuronal activity, resulting in insufficient oxygen and glucose supply to neurons and exacerbating the neuronal damage. In the later stages of stroke, during the process of neurovascular remodeling, the recovery of neurovascular coupling is often influenced by factors such as endothelial dysfunction, glial cell response, and inflammation [54,55,56,57]. Studies have shown that the recovery of neurovascular coupling in post-ischemic areas may be limited by the dysfunction of astrocytes and endothelial cells, preventing the cerebral blood flow from effectively returning to normal levels [58].

For hemorrhagic stroke, the rupture of blood vessels leads to hemodynamic changes in local brain regions, further damaging neurovascular coupling. Hemorrhagic stroke is often associated with severe fluctuations in the cerebral blood flow during the acute phase, which disrupts neurovascular coupling regulation and causes sustained brain tissue damage [59]. Research has indicated that endothelial damage, changes in the blood components, and local inflammatory responses often influence the local neurovascular coupling function following a hemorrhage.

### 3.4. Modeling Strategies to Explore Impaired Neurovascular Coupling

Computational modeling has become a powerful approach for understanding the complex dynamics of neurovascular coupling. Although most existing models focus on neurovascular coupling mechanisms under normal physiological conditions, these modeling frameworks also provide a strong foundation for exploring neurovascular coupling impairments in neurological and cerebrovascular diseases. A variety of computational models have been proposed to describe the coupling relationships among neural activity, the cerebral blood flow (CBF), and metabolism. For instance, Zheng et al. extended the traditional Windkessel model by redefining the parameters characterizing the pressure–volume relationship of blood vessels as state variables dependent on the rate of change in the cerebral blood volume (CBV). This modification enables the model to capture the vascular dynamics under varying physiological conditions and more accurately reflect the complex behavior of neurovascular coupling processes [60]. George employed computational fluid dynamics (CFD) models to simulate the blood flow within vessels through numerical methods, aiming to investigate the influence of the vascular structure and hemodynamics on neurovascular coupling [61]. Similarly, tuning parameters or modifying the structure of the existing models may allow their application in studying pathological neurovascular coupling impairments. For example, Khalid enhanced traditional CFD models by incorporating different blood viscosity profiles to better simulate the hemodynamics of cerebral aneurysms [62]. Amponsah proposed a fluid–structure interaction (FSI) model by integrating magnetic resonance imaging (MRI) data into the simulation, offering a means to predict the cerebrovascular damage in patients with traumatic brain injury [63].

### 3.5. Biomarkers Associated with Neurovascular Coupling Impairment

With the advancement of neuroimaging and molecular biology techniques, researchers have identified a range of biomarkers that may reflect the impairments in neurovascular coupling. These biomarkers not only help elucidate the underlying mechanisms of neurovascular coupling impairment but also offer new directions for early disease screening and progression monitoring. Neurofilament light chain (NFL), a well-established marker of axonal injury, shows elevated levels in the cerebrospinal fluid (CSF) and serum, indicating the extent of neuronal damage [64,65]. Neuron-specific enolase (NSE), a glycolytic enzyme expressed in neurons, is released into the CSF following neuronal injury. Elevated NSE concentrations are often closely associated with impaired neuronal function and abnormalities in the neurovascular coupling-related pathways [66]. Moreover, glial fibrillary acidic protein (GFAP), the major intermediate filament protein of astrocytes, is released when brain tissue is damaged. Increased levels of GFAP in the CSF and serum are indicative of neuroinflammation and injury [67]. Biomarkers such as NFL, NSE, and GFAP provide a crucial foundation for the development of non-invasive, multimodal tools for the clinical screening and subtyping of neurovascular coupling impairment.

## 4. Neural Activity and Blood Flow Measurement Methods

Neurovascular coupling reflects the metabolic demands of neural activity and serves as an important window for studying brain function and neuroregulatory mechanisms. Blood flow monitoring technologies based on the principles of neurovascular coupling have been widely applied in basic neuroscience research and clinical diagnostics, including the functional imaging of cognitive tasks, hemodynamic evaluation in stroke patients, and the early detection of neurodegenerative diseases. However, the significant discrepancy in temporal scales between neural activity and hemodynamic responses poses a major challenge in elucidating the mechanisms of neurovascular coupling. Neural activity, such as the generation of action potentials and synaptic transmission, typically occurs on a millisecond scale, enabling rapid responses to external stimuli or internal cognitive states. In contrast, associated hemodynamic responses—including the changes in CBF, CBV, and blood oxygen level dependent (BOLD) signals are considerably delayed, usually beginning to rise 1–2 s after stimulus onset, peaking around 4–6 s, and then gradually returning to baseline levels. Moreover, the regulation of cerebral blood flow is not governed by a single component but involves the coordinated interaction of multiple elements within the NVU, including excitatory and inhibitory neurons, astrocytes, smooth muscle cells, and endothelial cells. Single imaging or monitoring techniques are insufficient to capture the spatiotemporal characteristics of neurovascular coupling comprehensively [68,69,70]. Different measurement methods each have their advantages, and the current methods mainly rely on the synchronized monitoring of cerebral hemodynamics and neural activity. These methods include TCD, NIRS, fMRI, multimodal techniques, fUS, and miniaturized endoscopy (Table 2).

### 4.1. Transcranial Doppler

TCD is a non-invasive technique for detecting intracranial blood flow changes, enabling the real-time monitoring of the blood flow velocity in the intracranial arteries [81]. The principle of TCD is based on the Doppler effect, which refers to the change in the frequency of waves when there is relative motion between the wave source and the receiver. During cerebral hemodynamic response measurement, the TCD device emits ultrasound waves at a specific frequency through a probe, which are transmitted through the skull to the cerebral blood vessels. When the ultrasound waves encounter moving red blood cells in the bloodstream, they are reflected [82]. The frequency of the reflected ultrasound waves changes according to the direction of the blood flow. When the blood flows towards the probe, the reflected frequency increases, while a decrease in frequency occurs when the blood flows away from the probe. Since the magnitude of the frequency shift is proportional to the blood flow velocity, the TCD device calculates the blood flow velocity by analyzing the frequency shift of the reflected waves. This method allows for the real-time, non-invasive monitoring of the blood flow velocity changes in cerebral vessels [83,84].

As a non-invasive ultrasound technology, TCD plays an important role in basic neuroscience research. Marin recruited 14 healthy volunteers aged 20–26 to complete cognitive tasks activating the frontal lobe (Phonemic verbal fluency test, Stroop test, and Trail Making Test) and used TCD to simultaneously the record blood flow velocities in the left and right anterior cerebral arteries (ACA). The study found that all the cognitive tasks led to a significant increase in the blood flow velocity in both ACAs, with hemisphere dominance observed. During the Phonemic verbal fluency test and Trail Making Test, the right ACA showed significant dominance [85]. Lin recruited 21 healthy volunteers with an average age of 25.5 years to complete left hemisphere tasks (reading, calculation, color scaling) and right hemisphere tasks (facial recognition, spatial imagination, line direction), using TCD to record the blood flow velocity changes in the middle cerebral artery (MCA). The study found no significant difference in the average velocity between the left and right hemisphere tasks during rest and task execution. In the six tasks, except for the line direction task, the blood flow velocity acceleration on the left side was greater than on the right side. This difference was more prominent in the left-hemisphere tasks and smaller in the right-hemisphere tasks. These studies demonstrate that TCD can serve as a measurement tool for cognitive functions and psychological tasks in basic neuroscience research, promoting the development of this field [86].

Stefan, based on neuroscience research, applied TCD technology to clinical research. From March 2022 to December 2023, acute moderate-to-severe traumatic brain injury (TBI) patients who were admitted to the neurocritical care unit and underwent invasive intracranial pressure (ICP) measurement were studied. TCD was incorporated into the daily monitoring equipment. The clinical data analysis confirmed the importance and feasibility of TCD monitoring in multimodal neuro-monitoring [87]. Negad conducted 232 TCD tests on 58 children in a pediatric intensive care unit in Algeria. Each TCD test measured the systolic, diastolic, and mean blood flow velocities and calculated the pulsatility index (PI). The ability of TCD to detect intracranial hypertension was assessed, and the correlation between the ICP and TCD values in TBI children was examined. The study found that TCD is particularly suitable for monitoring post-traumatic brain injury and for confirming brain death by recording the cessation of cerebral circulation [88].

### 4.2. Near-Infrared Spectroscopy

NIRS is an optical imaging technique based on the penetration of near-infrared light through biological tissues, which is selectively absorbed by oxyhemoglobin (HbO) and deoxyhemoglobin (HbR). It is widely used to measure the changes in oxyhemoglobin concentration in the cortical region of the brain, thereby indirectly assessing the neurovascular coupling function. Specifically, NIRS devices emit near-infrared light at specific wavelengths, allowing it to penetrate biological tissues and interact with the molecules within the tissue through scattering, absorption, and other processes. Since HbO and HbR exhibit distinct light absorption properties at different wavelengths, NIRS can measure the oxygenation levels in tissues based on these properties. HbO absorbs light primarily around 760 nm, while HbR absorbs more light around 850 nm. By accurately measuring the changes in the reflected or transmitted light intensity, NIRS can estimate the concentrations of HbO and HbR, providing the dynamic monitoring of local cerebral oxygenation [89,90,91,92]. This feature makes NIRS an important tool for studying the changes in the brain blood flow, oxygenation status, and the relationship between the neural activity and blood flow, with a unique advantage in neurovascular coupling research [75,93,94].

Joaquín explored the application of NIRS in primate brain cognitive neuroscience, using an adult male macaque monkey as the subject, training it to complete spatial delayed response (DR) and non-spatial delayed match-to-sample (DMS) tasks. NIRS signals and field potential signals were recorded simultaneously from the posterior parietal cortex and prefrontal cortex of the monkey. The study found that during working memory, HbO_2_ increased while HbR decreased, and the negative potential in the prefrontal cortex during working memory was more significant than in the parietal cortex. This suggests that NIRS is an effective tool for studying the neurovascular coupling during cognitive processes in primates, providing a foundation for further research [95]. Paola designed a functional near-infrared spectroscopy imaging device (fNIRS) based on traditional NIRS equipment to measure the blood oxygen level changes in the brain cortex. This device eliminated the traditional optical fiber connection and adopted a portable design, allowing subjects to freely move in natural environments while recording the blood oxygen level changes in the brain cortex [96].

Luciana applied NIRS in clinical practice research, monitoring the blood oxygen level changes in neonates in the neonatal intensive care unit (NICU) of a university hospital in Brazil. The study evaluated the applicability of NIRS and demonstrated its potential as a continuous monitoring method for high-risk neonates [97]. Chen studied 47 congenitally deaf children with an average age of 35 months who underwent cochlear implantation surgery. Using NIRS, the brain blood flow dynamics were recorded in response to four different auditory conditions (natural speech, instrumental music, multi-speaker noise, and speech in noise). The recorded signals were used to build a model for predicting the rehabilitation outcomes of congenitally deaf children after cochlear implantation [98].

### 4.3. Functional Magnetic Resonance Imaging

fMRI is a non-invasive brain imaging technique based on MRI, primarily used to study brain activity patterns and hemodynamic responses. It relies on the BOLD signal, using the changes in the blood oxygen levels in different regions of the brain to infer the neural activity. In fMRI, the BOLD signal variations associated with blood oxygenation reflect the local neural activity. When the neuronal activity increases in a specific brain region, there is an increased demand for oxygen and nutrients to sustain this activity, leading to a rapid increase in the local blood flow, resulting in a rise in HbO and a decrease in HbR in that region [73,99,100,101,102]. This change is reflected as an alteration in the signal intensity on MRI images. fMRI captures consecutive brain images through multiple rapid scans, which display the changes in brain activity at different time points. During each scan, fMRI measures the BOLD signal in various brain regions and tracks the neural activity changes through time series analysis [103,104,105,106].

Li used fMRI technology to observe the impact of the task difficulty on neural representation. The participants were informed in advance whether the task would be difficult or simple, and fMRI was used to record the brain activity under different task difficulties. The study found that during difficult tasks, the frontal cortical activity was more intense, and the visual cortex had a higher decoding accuracy for memory targets. Increasing task difficulty led to more cognitive effort, affecting the neural representation in working memory [107].

fMRI has not only had a significant impact on cognitive neuroscience but also plays an increasingly important role in clinical neuroimaging. fMRI can guide neurosurgeons to avoid damage to critical brain tissue, thereby reducing the risk of new clinical deficits post-surgery [108]. The brain activity patterns in patients with depression or schizophrenia show significant differences from those in healthy individuals. Analyzing the nonlinear dynamics in fMRI data can help better understand the pathological mechanisms of mental disorders [109]. fMRI can also be used in the assessment of disorders of consciousness (DOC). Damiani conducted research on patients with DOC after coma, performing initial fMRI assessments during the acute phase, followed by further evaluations to analyze the relationship between these assessments and patient prognosis [108].

### 4.4. Multimodal Techniques

Single neuroactivity and blood flow monitoring technologies are often limited by their resolution and applicable range, making it difficult to analyze the dynamic characteristics of neurovascular coupling fully. The advantage of multimodal fusion lies in the complementarity of signals, significantly improving the ability to analyze neurovascular coupling. TCD-NIRS combines the advantage of TCD in monitoring the large vessels’ blood flow velocity with NIRS for measuring the local tissue blood oxygen changes, compensating for the limitations of TCD in local blood flow measurement while improving the accuracy of analyzing the hemodynamic regulation mechanisms [77,110,111,112,113]. Yang used the TCD-NIRS combination to explore the effects of caffeine on the brain’s hemodynamics and metabolism. By monitoring the MCA blood flow velocity with TCD and microvascular hemoglobin levels with NIRS, they analyzed the differences between the caffeine and non-caffeine groups, providing a comprehensive evaluation of how pharmacological and physiological changes affect the brain hemodynamics [114]. In clinical applications, Ding used TCD in 17 children with ventricular septal defects under one year old to record the MCA average blood flow and NIRS to record the tissue oxygen index, assessing the children’s carbon dioxide reactivity during cardiac surgery [115].

EEG-NIRS fusion relies on EEG to capture rapid neural activity changes and uses NIRS to measure the local brain region blood oxygen concentration changes, allowing for the synchronous analysis of the time dynamics between neural activity and the blood flow responses. Zama used this method to study the relationship between electrophysiological activity and hemodynamic response during motor preparation. The participants performed a button-pressing task at their own pace, with EEG monitoring the brain activity and NIRS recording the blood flow changes [116]. Chen applied this method to a mental concentration task during the Wisconsin Card Sorting Test (WCST), where NIRS measured the brain oxygenation functional changes, and EEG assessed the cortical connectivity and regional cortical activity, analyzing the brain activation state during the mental concentration task [117]. Tang designed an EEG-NIRS measurement system for clinical brain activity monitoring, with EEG and NIRS sensors arranged in an interlaced manner to maintain the same spatial resolution, using standard EEG electrodes and optical sensors of specific wavelengths to record the signals [118].

EEG-fMRI fusion methods further enhance the spatial resolution capabilities. EEG provides high temporal resolution neural signals, while fMRI uses BOLD signals with a high spatial resolution to localize the brain region activity, tracking the neural activity patterns across different brain regions and exploring the spatiotemporal evolution of brain functional networks. Wu applied EEG-fMRI to sleep analysis, using EEG as a neural activity indicator and fMRI as a measurement tool for the cerebrovascular response, recording simultaneous EEG-fMRI data during nighttime sleep in eight healthy adults. They analyzed the linear consistency changes of neurovascular relationships during non-rapid eye movement (NREM) sleep [119]. Wang further studied the dynamic changes in neurovascular coupling during sleep inertia, analyzing the coupling relationship between the EEG theta/beta ratio and spectral slope features with fMRI BOLD signals, showing the dynamic changes in neurovascular coupling during sleep inertia [120].

EEG-TCD technology can synchronize the detection of temporal changes between the neural activity and blood flow. In some cognitive tasks, neuronal activity occurs first, followed by a slight delay in blood flow changes. Combining the time resolution of TCD and EEG allows for the precise measurement of this time delay, providing further understanding of how the blood flow responds to changes in neural activity. Rojas used this method to conduct experiments in healthy volunteers, using TCD to assess the changes in the brain blood flow velocity and EEG to evaluate the neural activity changes, performing statistical complexity analysis of neurovascular coupling under cognitive stimulation [121]. Clinically, Yuan tested the feasibility of EEG-TCD in detecting neurovascular coupling in epileptic patients. By integrating real-time TCD and EEG signals into a workstation, they recorded the bilateral MCA blood flow velocity (CBFV) and EEG signals during seizures in 12 partial patients, analyzing the correlation between the EEG and CBFV results. Chen further applied EEG-TCD in predicting the risk of delayed cerebral ischemia (DCI) complications in patients after subarachnoid hemorrhage (SAH) surgery. The model combined TCD and EEG data from the patient’s hospitalization period to provide optimal DCI prediction [122].

## 5. Future Prospects

### 5.1. Basic Mechanisms

In the study of neurovascular coupling, the complexity of the underlying mechanisms remains an unresolved issue. Most existing research relies on static or single-time-point data, lacking a comprehensive understanding of the dynamic regulation processes of neurovascular coupling [123]. Furthermore, the differences in metabolic levels and brain structures in animal models limit the generalizability of the findings. To address these issues, future research should focus on integrating multimodal imaging techniques with single-cell electrophysiological recordings to analyze the spatiotemporal characteristics of neurovascular coupling. Additionally, in vitro organoid models could be utilized to simulate the neurovascular microenvironment, thereby revealing its signal transduction networks [42].

### 5.2. New Detection Methods

Currently, MRI and PET remain the core imaging techniques for studying neurovascular coupling. MRI, based on the principle of nuclear magnetic resonance, allows for the acquisition of multi-angle sectional images, such as axial, coronal, and sagittal views, which are crucial for comprehensively assessing the complex brain anatomy. On the other hand, PET tracks the distribution and metabolic dynamics of radioactive tracers to indirectly reflect the coupling relationship between the neural activity and blood flow response. Previous studies have shown that PET can be used to assess the changes in the brain blood flow before and after epileptic seizures, helping to reveal neurovascular coupling alterations caused by abnormal neural activity [124,125].

With the continuous advancement of imaging technologies, fUS technology has emerged as a promising tool for neurovascular coupling research due to its high spatiotemporal resolution and deep tissue penetration ability [126].The fUS is based on highly sensitive ultrasound Doppler imaging technology, which indirectly reflects the neural activity by detecting the changes in the microvascular blood flow. Its core principle involves the use of planar wave emission and coherent compounding techniques to generate whole-brain hemodynamic images that can penetrate the skull to image the deep brain regions. Compared to traditional ultrasound, fUS offers both high spatiotemporal resolution and deep detection advantages. Li applied this technology to an Alzheimer’s disease mouse model to investigate the neurovascular coupling impairment in the hippocampus [127]. Clinically, Soloukey explored the potential applications of fUS in awake brain surgery, discovering its ability to capture the task-induced functional cortical responses and the vascular differences between tumors and healthy tissues, providing rich anatomical and physiological information [128].

In addition to non-invasive or minimally invasive imaging methods, miniaturized endoscopy technology can be implanted into deep brain regions. It operates by labeling neurons or blood vessels with fluorescent dye, collecting the emitted signals, and reconstructing dynamic images. This technique offers ultra-high spatial resolution and millisecond-level temporal resolution. In a study conducted by Chen, 167 patients with trigeminal neuralgia were examined using a combination of surgical microscopy and miniaturized endoscopy to diagnose neurovascular compression. The findings indicated that this technique played a significant auxiliary role in the detection of neurovascular coupling, thereby improving the diagnostic accuracy [129].

### 5.3. Clinical Applications

Although the therapeutic strategies targeting neurovascular coupling remain in the exploratory stage, a range of emerging interventions have demonstrated considerable potential in both basic and clinical research. Neuromodulation techniques, as effective approaches for regulating the neurovascular coupling function, have shown promising therapeutic effects across various disease models. These techniques include deep brain stimulation (DBS), transcranial direct current stimulation (tDCS), and repetitive transcranial magnetic stimulation (rTMS). DBS involves the implantation of electrodes into the subthalamic nucleus to modulate abnormal neural electrical activity and improve the local cerebral perfusion. Studies have shown that DBS has become a standardized treatment option for patients with advanced Parkinson’s disease (PD) [130]. The tDCS, a non-invasive cortical stimulation technique, delivers a constant current to alter the resting membrane potential of neurons. Anodal stimulation typically increases the cortical excitability, while cathodal stimulation decreases it. Research indicates that tDCS can enhance the motor recovery following ischemic stroke [131]. The rTMS exerts therapeutic effects by applying pulsed magnetic fields to specific brain regions to modulate neuronal activity. Rezaei conducted a randomized trial involving 65 veterans with combat-related post-traumatic stress disorder (PTSD), who received bilateral rTMS, right-sided unilateral rTMS, or sham stimulation. The findings suggested that rTMS may be beneficial in treating PTSD symptoms [132].

In addition, pharmacological interventions offer viable avenues for modulating the neurovascular coupling function. For instance, the analogs of brain-derived neurotrophic factors (BDNF) have been shown to promote angiogenesis and neuronal repair in stroke models. The administration of BDNF analogs increases the vascular density and neuronal activity in the affected brain regions post-stroke, thereby facilitating neurovascular unit remodeling and accelerating the functional recovery. Meanwhile, anti-inflammatory therapy is also a critical direction for neurovascular coupling modulation. Anti-inflammatory agents, such as interleukin-1β antagonists, can suppress neuroinflammation and improve the neurovascular coupling function, slowing disease progression. Studies in Alzheimer’s disease models have demonstrated favorable therapeutic outcomes with such agents [133,134].

The combined application of neuromodulation techniques and vasoactive pharmacological agents provides a multidimensional, multi-targeted strategy for restoring impaired neurovascular coupling function. This integrated approach holds great promise for the treatment of various neurological disorders, including stroke, Parkinson’s disease, Alzheimer’s disease, and psychiatric conditions.

## 6. Conclusions

Here, we systematically review the complex, multi-level mechanisms underlying neurovascular coupling, highlighting how the precise coordination among neurons, glial cells, endothelial cells, smooth muscle cells, and pericytes ensure the optimal cerebral perfusion in response to neuronal activity. The disruption of these interactions, commonly observed in Alzheimer’s disease, stroke, and other neurological disorders, leads to impaired blood flow regulation, neuronal energy deficits, and accelerated neurodegeneration. The traditional monitoring techniques such as TCD, NIRS, fMRI, and EEG have established the foundational understanding of neurovascular coupling, while emerging technologies, including fUS, miniaturized endoscopy, and computational modeling, offer unprecedented spatial resolution and predictive capabilities. Promising biomarkers, including neurofilament light and glial fibrillary acidic protein, may enable the earlier detection and monitoring of neurovascular dysfunction. Furthermore, integrating multimodal neurovascular coupling assessment tools with intelligent diagnostics powered by machine learning could facilitate the real-time detection of subtle disturbances. Therapeutically, combining neuromodulation techniques (DBS, tDCS, rTMS) with targeted pharmacological interventions (BDNF mimetics, cytokine inhibitors) represents an innovative strategy to restore healthy neurovascular function and improve the clinical outcomes.

## Figures and Tables

**Table 1:** Neurovascular coupling in various clinical conditions.

Pathology	Impact on Neurovascular Coupling	Mechanism
Stroke	Impaired—particularly in the hemisphere of the insult	Brain edema, inflammation, impaired neurotransmission, and neuronal death impair normal neuronal activation. Also, the activation of non-specific brain structures further complicates interpretation [34,35,36].
Hypertension	Impaired	Elevated circulating angiotensin II leads to (1) activation of ATI receptors on the cerebral blood vessels, (2) increased oxidative stress which inhibits neuronal and astrocytic vascular dilators [37].
Autonomic Dysfunction	Impaired	Impaired blood pressure responses and the altered neurogenic regulation of neurovascular coupling [38].
High level	Impaired	Unknown persistent dysfunction of neurovascular coupling after restoring normal blood pressure [39].
Traumatic Brain Injury	Impaired	Neuronal death and astrocytic scar formation preclude the normal neurovascular response [40].
Alzheimer’s	Impaired	Hypercontractility (phenylephrine) of smooth muscle, increased basal, and the enhanced occurrence of spontaneous Ca^2+^ waves. Amyloid Beta also directly inhibits functional hyperemia by promoting oxidative stress, which inhibits neuronal and astrocytic vascular dilators [41].

**Table 2:** Neurovascular coupling measurement methods.

Method	Principle	Temporal Resolution	Spatial Resolution	Advantages	Limitations
Transcranial Doppler (TCD)	Based on the Doppler effect, it monitors cerebral artery blood flow changes to assess the cerebral blood flow changes.	High (approximately 1–10 ms)	Low (>10 mm; no anatomical localization capability)	High temporal resolution and real-time capture of blood flow velocity changes.	Only measures the blood flow velocity in large brain arteries, not directly assessing brain tissue perfusion; affected by the skull thickness [71].
Near-Infrared Spectroscopy (NIRS)	Optical imaging techniques measure the changes in oxygenated (HbO) and deoxygenated hemoglobin (HbR) concentrations in the brain.	Moderate (approximately 100 ms–1 s)	Moderate to low (approximately 1–3 cm; limited to the cortex)	High temporal resolution and real-time monitoring of blood flow changes induced by neural activity provide information on the local oxygenation status.	Limited to the cortex, cannot measure deep brain regions; affected by skin and skull [72].
Functional Magnetic Resonance Imaging (fMRI)	Relies on the BOLD signal to detect the changes in the ratio of oxygenated to deoxygenated hemoglobin in brain tissue.	Low (approximately 1–2 s)	High (approximately 1–3 mm)	High spatial resolution and full-brain coverage, ideal for studying neurovascular coupling and task-related cerebral blood flow changes.	At lower temporal resolution, the BOLD signal indirectly reflects neural activity and may be influenced by various factors [73].
Multimodal Techniques (EEG combined with TCD/NIRS/fMRI)	EEG is combined with other brain blood flow methods (TCD, NIRS, fMRI) to record the neural activity and blood flow changes simultaneously.	Complementary	Complementary	Provides synchronized information on neural activity and blood flow regulation, enhancing the comprehensive assessment of neurovascular coupling.	Synchronization issues between different techniques and increased complexity in data processing and analysis [74,75,76,77].
Functional Ultrasound (fUS)	Measures cerebral blood volume (CBV) changes via ultrafast Doppler imaging.	High (<100 ms)	High (approximately 100 µm)	High spatial and temporal resolution; sensitive to deep and microvascular flow.	Requires a cranial window or acoustic access; relatively new in humans [78].
Miniaturized Endoscopy	Uses small optical probes to directly visualize fluorescence or hemodynamic signals in the deep brain.	High (<100 ms)	High (approximately 100 µm)	Allows deep-brain imaging in freely moving animals; high-resolution access to specific structures.	Invasive; limited field of view; primarily animal studies [79,80].
